# Decreased levels of discomfort in repeatedly handled mice during experimental procedures, assessed by facial expressions

**DOI:** 10.3389/fnbeh.2023.1109886

**Published:** 2023-02-02

**Authors:** Julia Swan, Scott Boyer, Karolina Westlund, Camilla Bengtsson, Gunnar Nordahl, Elin Törnqvist

**Affiliations:** ^1^Research Unit of Biomedicine, Department of Pharmacology and Toxicology, University of Oulu, Oulu, Finland; ^2^Biocenter Oulu, University of Oulu, Oulu, Finland; ^3^Chemotargets SL, Barcelona, Spain; ^4^Global Safety Assessment, AstraZeneca R&D, Södertälje, Sweden; ^5^ILLIS Animal Behaviour Courses, Stockholm, Sweden; ^6^Independant Consultant, Strömsund, Sweden; ^7^Sweden Operations, AstraZeneca, Södertälje, Sweden; ^8^Department of Animal Health and Antimicrobial Strategies, Swedish National Veterinary Institute (SVA), Uppsala, Sweden; ^9^Institute of Environmental Medicine, Karolinska Institutet, Solna, Sweden

**Keywords:** 3R, mouse grimace scale, refinement, repeated handling, habituation, sex differences

## Abstract

Mice are the most commonly used laboratory animal, yet there are limited studies which investigate the effects of repeated handling on their welfare and scientific outcomes. Furthermore, simple methods to evaluate distress in mice are lacking, and specialized behavioral or biochemical tests are often required. Here, two groups of CD1 mice were exposed to either traditional laboratory handling methods or a training protocol with cup lifting for 3 and 5 weeks. The training protocol was designed to habituate the mice to the procedures involved in subcutaneous injection, e.g., removal from the cage, skin pinch. This protocol was followed by two common research procedures: subcutaneous injection and tail vein blood sampling. Two training sessions and the procedures (subcutaneous injection and blood sampling) were video recorded. The mouse facial expressions were then scored, focusing on the ear and eye categories of the mouse grimace scale. Using this assessment method, trained mice expressed less distress than the control mice during subcutaneous injection. Mice trained for subcutaneous injection also had reduced facial scores during blood sampling. We found a clear sex difference as female mice responded to training faster than the male mice, they also had lower facial scores than the male mice when trained. The ear score appeared to be a more sensitive measure of distress than the eye score, which may be more indicative of pain. In conclusion, training is an important refinement method to reduce distress in mice during common laboratory procedures and this can best be assessed using the ear score of the mouse grimace scale.

## 1. Introduction

Laboratory animal welfare is influenced by handling procedures. This includes housing and routines which range from arriving at the research facility until the animals are sacrificed at the end of the study. Stress and suffering may compromise the animals’ health and welfare, as well as introduce problematic, confounding factors that may interfere with the interpretation of biomedical results (Ghosal et al., 2015; Ono et al., 2016). The development of methods and activities for refinement is therefore important to reduce stress before, during and after the animal experiment. Refinement activities are often associated with improved housing conditions and environmental enrichment (Baumans and Van Loo, 2013). This can include the usage of non-aversive animal handling methods (Hurst and West, 2010) and animal training (Westlund, 2015).

Stress has the potential to impact physiological responses in two ways: increasing variability (Koolhaas et al., 2010; Fridgeirsdottir et al., 2014; Nakamura and Suzuki, 2018) or acting as a confounding factor (Rowan, 1990; Strekalova et al., 2005; Ghosal et al., 2015; Ono et al., 2016). Consequently, proper acclimation (adaptation to environmental conditions), including handling and sampling procedures, is crucial to safeguard the quality of the data from acute stress reactions.

In addition to improved housing and enrichment, gentle handling, and training should be employed as a refinement protocol before any experimental procedure is performed. Animal training or structured human-animal interactions reduces laboratory animal stress and has been effective for numerous lab animal species. For example, in primates, training has been shown to reduce fear and associated stress responses (Westlund, 2015) and, in rats, tickling reduces the stress of repeated intraperitoneal injections (Cloutier et al., 2014).

However, there have been limited studies on the refinement effects of mouse training, although they are the most used laboratory animal (with 5.5 million being used in EU, constituting approximately 52.5% of laboratory animals) (European Commission, 2022). Hurst and West (2010) compared the anxiety levels induced by differing mouse handling methods. Mice lifted by their tails exhibited high anxiety and handler aversion compared to those lifted in their shelters/tubes, or in an open hand (cup) (Hurst and West, 2010). Gouveia and Hurst (2019) later investigated the duration and handling frequency needed to familiarize mice with these different lifting methods (tail, tube, and cup lifting). More handling sessions were needed to habituate mice to be cupped on an open hand, compared with tube lifting (Gouveia and Hurst, 2019). Moreover, a strong handler aversion was observed after only a short duration and frequency of tail lifting. The positive effects of frequent handling with non-aversive techniques (tube lifting for instance) were not affected by scuffing or subcutaneous injections (Gouveia and Hurst, 2019). This suggests that these handling techniques have practical application in laboratory settings where mice are frequently required to be restrained for routine procedures.

The refinement aspect of the 3Rs concept is defined as a method which alleviates or minimizes potential pain, suffering, and distress, and which enhances animal well-being (MacArthur Clark, 2018). This study focuses on the refinement component of 3R’s. Understanding mouse behavior and signs of welfare vs. distress is pertinent to evaluate efforts aiming at refinement (Brown et al., 2006). General welfare assessment protocols for scoring animal suffering have been used to measure the effects of refinement strategies for research animals (Hawkins et al., 2011), and for specific experimental animal models, e.g., experimental autoimmune encephalomyelitis (Wolfensohn et al., 2013). In 2010, Langford et al. (2010) published the Mouse Grimace Scale (MGS) for assessment of facial expressions of pain in the laboratory mouse. This highly cited method, not only allows identification of the degree of pain in mice, but also shows that mice express feelings of pain using facial expressions, just like humans (Leach et al., 2012). Could mice also reflect feelings of distress in their facial expressions?

In this study we investigate effects of mouse training during acclimation by scoring the ear and eye appearance according to the MGS (Langford et al., 2010) during subsequent injection and blood sampling. We hypothesize that trained mice are less stressed than mice handled using traditional handling methods. In addition, we assess the usefulness of the MGS during non-painful as well as painful handling procedures. Here, we adapted this scoring method to assess stress and discomfort in mice, by focusing on the ear and eye categories of the scoring system.

## 2. Materials and methods

### 2.1. Animals and housing

Twenty male and 20 female, 4-week-old, CD1 mice were purchased from Charles River where they were tail lifted. The mice were randomized into groups of two or three per cage. Randomization was done by placing the first mouse lifted from their travel cage in group 1, the second in group 2, the third in group 1, etc. This was done within each gender group so that there were 20 animals per group (10 males and 10 females). The mice were housed in Macrolon 3H cages with bedding (Aspen bedding), nesting material (happy mats and sizzle nest) and a cardboard house and tunnel. They were provided standard chow and tap water ad libitum. Their cages were changed once weekly and they were maintained with a 12 h light/dark cycle, temperature between 19 and 21°C, and humidity of 40–70%. The study was approved by the Swedish Research Animal Ethics Committee (Stockholms södra djurförsöksetiska nämnd).

### 2.2. Study design

**FIGURE 1:**
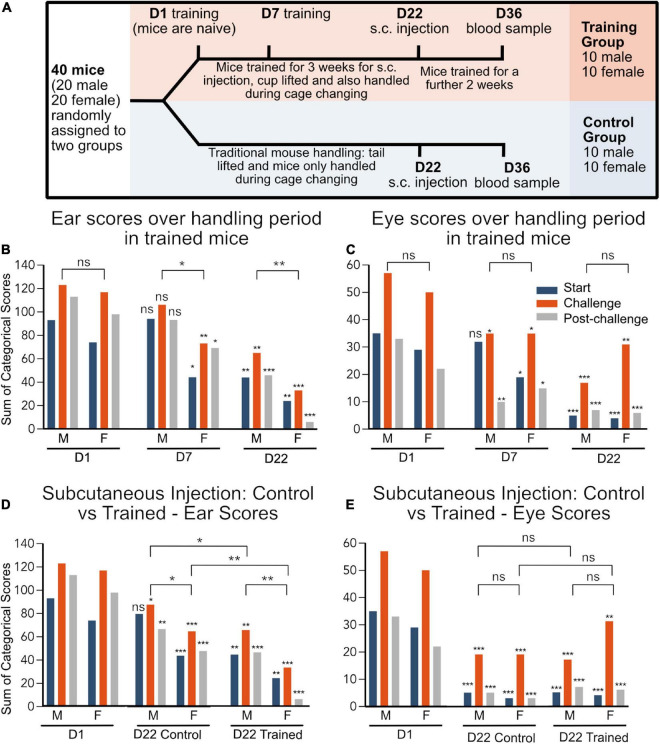
**(A)** Experimental timeline. D1 of training is used as a baseline as this is the first handling session. **(B–E)** Data are represented as the sum of categorical scores: the sum of facial scores from all evaluators (7 evaluators for ear and 6 evaluators for eye) for all mice in each treatment group (*n* = 10). Maximum possible score for ear: 140, maximum possible score for eye: 120. *p < 0.05, **p < 0.01, ***p < 0.001. Significance indicated directly above each bar indicates comparison to Day 1 category of the same sex group and handling observation, e.g., start, challenge, and post-challenge. Comparisons between groups for sex difference and training effect (combined start, challenge, and post-challenge scores) are indicated by lines above the bars. D1, day 1; D22, day 22 and corresponds to s.c. injection.

In an experiment setting, the facial expressions of trained, cup lifted (test group) and non-trained, tail lifted (control group) mice were compared during common research procedures; namely subcutaneous (s.c.) injection and tail vein blood sampling (Figure 1A). The mice were handled by the same female technician for all procedures and all staff interacting with the mice were also female. The group allocation was known by the animal handlers during conduction of the experiment, i.e., in training sessions as well as s.c. injection and blood sampling.

The control group was handled using traditional handling techniques. This means that the mice were acclimated for 1 week and had daily observations through the cage, followed by a thorough examination once weekly. This was accompanied by regular cage changes. These mice were always lifted by their tails, including during cage changes.

The test group was acclimated for 1 week, thereafter, they were trained according to a schedule. In the schedule, mice were trained five times a week for 3 weeks (before s.c. injection) and then trained for a further 2 weeks (a total of 5 weeks of training) before blood sampling. The training procedure was designed to prepare the mice for dosing and sampling procedures commonly used in toxicological studies: the mice were cupped in the technician’s hand and put on a soft piece of fabric/soft pad placed on the procedure table. The skin was lifted/pinched, simulating skin lifting at subcutaneous injection, at four possible injection sites and the mouse was cupped in the hand again and transferred back to the cage (Supplementary Video 1). Each session lasted approximately 8–10 seconds. The mice were always lifted by cupping, including during cage changes.

After 3 weeks of training, both groups were injected subcutaneously, but no substance was administered. The test group was then trained for a further 2 weeks (a total of 5 weeks of training) and on day 36, mice from both groups had blood sampled *via* the tail vein (Figure 1A). The subcutaneous injections and blood sampling *via* the tail vein were performed using a non-restrained method in all groups. Here, the mouse was placed on a soft piece of fabric on the lab bench where all training and experimental procedures were performed. The injections and sampling were done in groups of 10 according to the treatment groups.

Each animal in the test group was filmed at the first and seventh day of training, and each mouse from both groups (test and control) were filmed at day 22 and day 36, when injected subcutaneously and blood was sampled intravenously, respectively.

### 2.3. Scoring protocol

Ear scoring and eye scoring were performed using a three grade scale from the MGS (Langford et al., 2010): 0 = normal ear position and eye appearance (Supplementary Videos 1, 2), 1 = slightly changed ear position and slightly altered eye appearance (Supplementary Videos 3, 4) and 2 = totally changed ear position and altered eye appearance (Supplementary Videos 5, 6). Example videos of blood sampling can be seen in Supplementary Video 7 (control) and Supplementary Video 8 (trained).

Each animal was scored at three time points in each film: before, during and after the challenge (pinch/injection/needle puncture). The scoring was done by seven evaluators, all of them experienced animal technicians working with mice in toxicity studies (see Supplementary Table 1 for an example of the scoring sheet).

The films were randomized, all identifiers removed and shown to all evaluators at the same occasion. All films were displayed twice, and evaluators registered their score on separate sheets without conferring with each other. Ear and eye scores were recorded in the same viewing session. The evaluators scored in total 120 films (80 films from the trained group and 40 from the non-trained group). In addition, as an internal validation of the scoring procedure, 16 of these films were randomly selected and displayed twice, once in the beginning of the session and a second time at the end to evaluate the scoring stability over the day. The evaluators’ individual scoring was also compared.

### 2.4. Statistics

Each mouse was considered a single experimental unit and no unit or data point was excluded during the analysis, thus the sample size was 10, unless stated otherwise.

The results of both ear and eye scores were analyzed separately and grouped by sex, treatment (and day) and by handling event (pre-challenge, challenge, and post-challenge). Comparisons between groups used individual scores from the evaluators and were treated as three-level ordinal variables (Score 0, 1, 2). Group comparisons were performed using Ordinal Logistic Regression (OLR) (Venables and Ripley, 2002) using the MASS function^1^ in RStudio (2022.07.1 Build 554). Significance values, expressed as *p*-values, were derived from an ANOVA with a *post hoc* Chi-squared test comparing the null-hypothesis. All regression coefficients from the OLR are set to zero to the OLR derived from the group comparisons. The observed *p*-values are reported as not significant (ns) (*p* ≥ 0.05) or significant at *p* < 0.05, *p* < 0.01, and *p* < 0.001.

For the evaluation of training effect on specific responses, pre-challenge, challenge, and post-challenge responses were compared to their day 1, within-sex response. For the evaluation of sex effects and overall training effects all within-sex scores (pre-challenge, challenge, and post-challenge) were grouped for each training/evaluation occasion (day). Overall training effects and sex differences were evaluated using these grouped scores.

Evaluation of the uniformity of observer scores (*n* = 7 for ear and *n* = 6 for eye) was performed using OLR in RStudio (2022.07.1 Build 554) with a chi-squared statistic with the group of scores for each observer across all test conditions grouped and compared to all other observers.

To control for observer scoring consistency, 16 films were displayed twice, to evaluate the scoring stability over the day (*n* = 16). Normality was evaluated using the Shapiro–Wilk test. The scores recorded at the start of the session were compared to those scored at the end of the session using a repeated measures *t*-test (normal distribution) or a repeated measures Mann Whitney test (non-normal distribution). This was conducted in GraphPad Prism Version 9.

## 3. Results

### 3.1. Trained mice display less discomfort than non-trained mice

At day 22, the mice had a s.c. injection, the facial scores were recorded and, later, scored. Both male and female trained mice had lower ear and eye scores, when compared to the first day of training for this procedure (Figures 1B, C). Furthermore, when the facial expressions of the control vs. trained group were compared at D22, trained mice had lower ear scores than the control mice, showing reduced discomfort (Figure 1D). However, there were no differences in eye score between trained and control mice (Figure 1E).

The facial expressions of the control group were also compared with that of the training group’s facial scores on day 1. Facial scores on day 1 provide a baseline for all the mice as this was the first time that the trained mice were handled, thus at this point, they are considered naïve (Figure 1A). Interestingly, at day 22, the untrained mice had lower ear and eye scores when compared to day 1 of the training group (Figures 1D, E). This suggests that, despite not being trained for handling, the mice became habituated to their environment over the 22-day period, which also impacted the facial scores and level of discomfort during s.c. injection.

**FIGURE 2:**
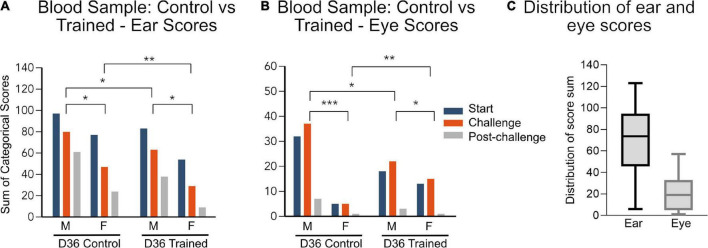
**(A,B)** Data are represented as the sum of categorical scores: the sum of facial scores from all evaluators (7 evaluators for ear and 6 evaluators for eye) for all mice in each treatment group (*n* = 10). Maximum possible score for ear: 140, maximum possible score for eye: 120. Comparisons between groups were performed using grouped data for each sex and training/control group. Significant differences between groups are indicated by lines above the bars, *p < 0.05, **p < 0.01, ***p < 0.001. D1, day 1; D36, day 36 and corresponds to tail vein blood sample. **(C)** Distribution of all the ear and eye scores, combining treatment group and sex.

On day 36, the mice had blood sampled from their tail vein–a procedure for which neither group was specifically trained. The changes in facial expression seen here in the trained group is a result of habituation to general handling. Here, trained mice had lower ear scores than control mice (Figure 2A) and trained male mice had lower eye scores compared to untrained male mice (Figure 2B). However, unexpectedly, the female eye score from the trained mice was higher than that of the untrained mice (Figure 2B).

### 3.2. Ear scoring was more sensitive than eye scoring, in this study

Data are represented in the graphs as the sum of categorical scores: the sum of facial scores from all evaluators (7 evaluators for ear and 6 evaluators for eye) for all mice in each treatment group (*n* = 10) (mean score and SD for individual groups in Supplementary Table 2). The maximum possible score is 140 and 120 for the ear and eye, respectively. The maximum recorded ear score was 123 and the maximum recorded eye score was 57. There was also a difference in distribution of the data with a minimum of 6 for the ear and 1 for the eye score. Furthermore, the mean ear score was 73.5 for the ear and 19 for the eye. The larger range of distribution (Figure 2C) in the ear score, compared to the eye score, suggests that the ear score may be a more sensitive measure of discomfort than the eye scores overall. However, in response to challenge, the eye scores appear to be a more sensitive measure of pain/perceived pain (Figures 1C, E, 2B).

### 3.3. Females had lower ear scores after training, compared to males

There was no difference in baseline ear and eye scores in male and female mice at day 1. However, after 7 days of training, mimicking subcutaneous injection, there was a reduction in ear score in females but not in males (Figure 1B). Compared to day 1, both male and female mice had reduced challenge and post-challenge eye scores with only the females having reduced start eye scores (Figure 1C).

Only at the subcutaneous injection, after 22 days of training, was the ear score reduced in the male mice (Figure 1B), suggesting that males may require more training sessions than females to reach the same level of habituation to handling. On the other hand, after 22 of training, the eye scores were reduced in in both males and females at all time points (start, challenge and post-challenge) (Figure 1C).

In both the trained and untrained group, the females had overall lower scores in both the ear and eye score categories compared to the males. However, this was only statistically significant when evaluated using the ear scores.

### 3.4. There was no significant difference between scores performed by different test persons

Evaluation of the distribution of scores between observers across all films in this study demonstrated that there does not appear to be any significant difference in the distribution of scores from any one observer and the remaining group of observers or between individual observers.

In addition, there was no significant change in the scores for the 16 films evaluated at the beginning of the day, when compared to the end of the day (an indication of score stability) for the ear score. However, the eye scores recorded at the end of the session were lower (mean 8.3, max 17, min 1) compared to the beginning of the session (mean 10.4, max 24, min 1) (Supplementary Figure 1).

## 4. Discussion

### 4.1. Habituated animals are less stressed

In the present study, trained mice were handled five times per week for 3–5 weeks, and during the 8–10 second handling sessions, the handler mimicked procedures associated with a subcutaneous injection: partial restraint by the base of the tail, and four skin lifts on both shoulders and flanks. The control group was handled according to traditional handling procedures, e.g., tail lifted and only handled during cage changing. Trained mice expressed less distress than the control mice during experimental procedures, when evaluated using the orbital tightening and ear position categories of the MGS. However, the ear score was the most sensitive measurement of distress.

Animals may be expected to either sensitize (show an increased stress response over time) or habituate (show a decreased stress response) to a stimulus (McSweeney and Murphy, 2009). Whether one or the other occurs depends on how the introduction to the potentially aversive stimulus is carried out, as well as how aversive it is. In the current study, the aversiveness of the procedure was reduced by lifting the mice in cupped hands, as well as minimizing restraint: the animal was placed on a soft piece of fabric–a possible explanation as to why the animals habituated rather than sensitized in the present study.

Acclimation to certain experimental procedures or situations has previously been shown (Westlund, 2015; Kärrberg et al., 2016; Lindhardt et al., 2022). For example, stress-associated weight change in mice was avoided by acclimating mice to the oral gavage procedure, either by sham gavage or by restraint (Kärrberg et al., 2016). However, the training can be stressful. Body weight loss was observed during the acclimation compared to the untrained mice, which indicates that the training, including restraint, was stressful. Restraint for injections and blood sampling triggers stress in mice (Meijer et al., 2006). Consequently, habituation to general handling should be the goal of the training sessions, not necessarily training for the specific procedure.

Acclimation to this particular short training procedure is expected to generalize to a variety of different experimental procedures, especially procedures which allow for the mice to be in contact with the fabric/soft pad. During the training sessions, the mice became accustomed to a sequence of events. Firstly, they were enclosed by a gloved hand and removed from the cage–an important step expected to occur with any type of procedure. Within a few seconds, they were placed on a soft fabric pad on the procedure table. In this situation, the tactile, visual, and olfactory sensations of the environment may dominate and, to some extent, even overshadow the subsequent handling/skin lifting/pinching. Finally, after a few seconds, the animals were returned to their cage. As a result, we expect that the mice would have learned that the whole experience (being enclosed within gloved hands, sitting on a soft pad for a few moments and experiencing a plethora of sensations, returning to their home cage) was highly predictable and therefore less stressful over time. Consequently, if we were to keep most of the sequence intact, then we would expect the calming effect of the training to generalize to several types of experimental procedures in the trained mice. This generalized effect of the handling was demonstrated by the reduced stress during blood sampling on habituated animals compared to controls. Such carry-over effects of handling for procedures other than those the animals were specifically trained for has already been previously demonstrated (Gouveia and Hurst, 2019; Marcotte et al., 2021).

Mice have also been successfully trained using intricate positive reinforcement protocols (Leidinger et al., 2017). These require time and trainer skill; in a clicker training study by Leidinger et al. (2017), each training session was 5 min, and the training followed a 38-step training manual. This illustrates that training using operant procedures requires some skill from the handler. Most animal technicians, although skilled in technical laboratory animal procedures such as blood sampling, might not have the training to successfully shape operant responses using positive reinforcement and successive approximations of desired responses (shaping).

However, the acclimation procedure used in the current study was simply exposing the animal to the future experimental procedure multiple times before the experiment started, albeit replacing the final injection with four skin lifts. Furthermore, each training session was only 8–10 seconds long. Two unique additions in the current study that may not be in use in all facilities was to (a) lift the mouse in a cupped hand, and (b) put the mouse on a soft pad rather than use restraint during the experimental procedure. Both techniques require little training to master for the handler, and effectively reduce stress for the animal.

This technique could be further refined by combining systematic desensitization (gradually introducing the handling/stimulus over several training sessions) with counter conditioning (immediately following each training session with access to a desired resource, such as food treats) to prevent and diminish fear and stress. This approach may diminish the stress scores observed in the current study during the initial days of acclimation. Further investigation to reduce the training time is also required for training to be practical and economically viable. Recently, a 3-day training technique was developed to habituate mice to handling (Marcotte et al., 2021). This method involves increased interaction with an individual mouse over a period of 3 days, with different milestones to reach before moving to the next step. This method could be tested, using facial expressions to determine the optimal number of training sessions needed per strain and sex.

### 4.2. The mouse grimace scale measures not only pain, but also distress/fear/discomfort

In this study, the positive effects of training laboratory mice were successfully assessed by the ear position of the MGS. The ear score was a more sensitive measure of discomfort, compared to the eye score. Furthermore, the ear score was more stable over the duration of the scoring session, when evaluated using the internal validation test. However, the eye score may be a more sensitive marker of pain or perceived pain. This is evident by the clear peak in eye scores, but not ear scores, during s.c. injections and pinching (Figures 1C, E).

Interestingly, there was also a difference in peak score patterns between the s.c. injection and tail vein blood sampling. During the training sessions and s.c. injection, the ear and eye scores peaked during the challenge. However, during the blood sampling on day 36, the ear score peaked before the challenge, and the eye score peaked at the challenge. This could be attributed to slight differences in handling the mice in the different procedures. During training and s.c. injection, the mouse is briefly held at the tail base, quickly followed by an injection and or pinch. In contrast, during the blood sampling, the mouse is restrained by the tail base for a longer period, during which the tail vein is identified before puncture. This longer duration of restraint may cause additional distress which exceeds that of skin puncture. If the ear is a more sensitive measurement of discomfort, and not pain, this would explain why the peak score was before the challenge and the eye score still peaked during the challenge.

Changes in facial expression is an interspecies indicator of pain and emotional status (Zych and Gogolla, 2021) and facial scoring systems in numerous animal species have been developed to improve animal welfare (Boissy et al., 2011; Bellegarde et al., 2017; Marcet Rius et al., 2018; Lambert and Carder, 2019). The MGS system was initially developed to assess pain in mice (Langford et al., 2010) but, here, the orbital tightening and ear position categories of the MGS were used to assess fear and distress.

The ear is the most mobile part of the face in many animal species and easily assessed. Particular attention to ear position has been given to evaluate emotional states of farm animals. For example, Boissy et al. (2011) found that negative emotional states in sheep were associated with the ears being pulled back and, in positive states, the ears were pulled forward. Similar associations between emotional state and ear position have been found in goats (Bellegarde et al., 2017), cows (Lambert and Carder, 2019), and pigs (Marcet Rius et al., 2018). However, there has been limited investigation into the association between facial expression and emotional states of laboratory animals. Finlayson et al. (2016) used the rat grimace score in combination with other measurements to assess the effect of positive interaction such as tickling on the facial expression of rats. They found that the positive treatment was associated with a pinker ear color and wider ear angle (Finlayson et al., 2016).

In mice, Defensor et al. (2012) used the MGS to evaluate their emotional state in different contexts of stress; such as predator stress, intruder stress and from the discomfort due to whisker stimulation with a brush, etc. Some components of the MGS, such as the ear position and orbital tightening were strongly associated with direct contact or potential for contact, e.g., when the mouse whiskers were directly touched with a brush, two unfamiliar mice were placed opposite each other across a barrier, or an intruder mouse was placed in the home cage. In contrast, the nose bulge and cheek swell scores were increased more with potential exposure to a predator, i.e., when exposed to the scent of a cat or exposure to a rat across a barrier. This supports our use of the orbital tightening and ear position score to assess mild to moderate stress during handling.

Dolensek et al. (2020) further investigated this link between facial expression and emotional state of mice using image analysis and machine-learning as an alternative to the MGS. They showed that facial expressions are reliable indicators of emotional states and confirmed this thorough optogenic stimulation of subregions and projections of the insular cortex. They also used optogenic stimulation to manipulate γ-aminobutyric acid–releasing neurons in the ventral pallidum, which process reward to pleasant stimuli. Although image analysis of facial expressions can provide an objective assessment of the mouse’s emotional state, it requires special equipment, expertise which can only be assessed post-handling.

Most methods used to evaluate the success of habituation/gentle-handling techniques on the mouse’s stress levels and welfare often require specialized behavioral or biochemical tests. For example, the elevated plus maze test, handler interaction tests, fecal or blood corticosteroid levels, etc. Here, by handlers focusing on the ear position categories of the MGS, they will be able to assess the response of the mouse to their handling/training during each interaction. This is thus a simple, cost-effective tool which can be used daily to evaluate the level of stress in mice to improve welfare.

### 4.3. Sex difference in response to training

Despite having the same baseline facial expression scores, trained, female mice had, overall, lower scores compared to the males in both the ear and eye score categories. They also had a significant reduction in facial scores after the first week of training, which was not seen in the male mice until day 22.

To improve rigor and translatability of preclinical research, many grant funding organizations now require applicants to include both male and female animals in the experimental design (Shansky and Murphy, 2021). This means that more female mice are expected to be used in preclinical research than in the past. As a result of these funding changes, there is also increasing evidence that male and female, animals and humans alike, respond differently to stress (Bangasser and Wicks, 2017). This evidence supports our findings that male mice respond differently to training compared to female mice.

However, the effects of sex on the number of training sessions required for habituation have not been reported. For example, in the 3-day training technique by Marcotte et al. (2021), habituated male C57BL/6 mice had increased voluntary interaction and decreased anxiety-like behaviors (measured by novelty-suppressed feeding and elevated plus maze), compared to the tail lifted groups. In females, training did not affect these measurements, but did result in decreased serum cortisol levels after 3 days of handling. However, the male and female groups used in that study were different ages, confounding these sex differences (Marcotte et al., 2021). In another study, testing a 14-day habituation protocol of C57Bl/6 mice to magnetic resonance imaging (MRI), male mice had a significant decrease in heart rate after 10 days of training, and females, after 11 days. Females had higher fecal corticosterone metabolite levels, compared to the males, suggesting that they were more stressed throughout the training protocol. However, there were no other indications that the females habituated to the MRI simulation at a different rate than the males (Lindhardt et al., 2022).

### 4.4. Future areas of research

Here, we show that the ear score, a component of the MGS, is a sensitive method to measure distress in mice. However, further studies are needed to refine this scoring system to focus on signs of distress, rather than pain. In addition, strain differences should also be investigated. Here, only female handlers worked with the mice and handler gender has been shown to influence the level of stress in mice (Sorge et al., 2014), consequently the effect of handler gender on the facial scores should also be studied.

A handful of studies have shown that the methods used to lift mice can affect endpoints used in biomedical (Ghosal et al., 2015; Ono et al., 2016) and behavioral (Novak et al., 2015; Gouveia and Hurst, 2017; Ueno et al., 2020) research. Consequently, a follow up study should assess the effects of training on biochemical parameters of interest in a biomedical study, e.g., severity of a disease model, measurement of metabolic parameters, etc.

### 4.5. Limitations

The required 3–5-week training duration may be prohibitively long and time consuming within certain fields. In future studies, the training duration could be considerably reduced as an improvement in facial scores, in females, was already seen on day seven. Perhaps, the facial scoring used in this study could be more widely adopted, when combined with the 3-day training protocol (Marcotte et al., 2021). Furthermore, there may have been some group effect as, for logistical reasons, mice were injected and sampled in their treatment groups. Further studies should aim to inject/sample the mice in mixed groups.

The mice were trained in the mornings, during their light cycle/rest period which may have effects on their sleep. This may be a factor which needs to be addressed in all future training protocols used for crepuscular, nocturnal animals. Training should be done at the beginning/end of the dark cycle or during the dark cycle, possibly using a reversed-light cycle or time-shift approach (Hawkins and Golledge, 2018). Although tested to evaluate sleep deprivation in mice, Longordo et al. (2011) did investigate the effects of 3 min disturbance of sleep in mice during the light phase, over 6-days. This is similar to what mice would experience during a training protocol. On all six handling days, there was approximately 25% reduction in resting time and serum cortisone levels were raised. Consequently, routine handling of mice during the light cycle could also introduce confounding effects in behavioral and biomedical studies and needs to be considered when establishing a training protocol (Hawkins and Golledge, 2018).

Lastly, this study investigated training in cup lifted mice and compared it to untrained tail lifted mice. Consequently, some of the changes seen in facial expressions between the two groups may not purely be due to training, but also be influenced by the handling method used.

## 5. Conclusion/Summary

Habituating mice, through training, to common laboratory procedures for 8–10 seconds during the acclimation period significantly reduces stress. This study was conducted using CD1 mice as they are a general-purpose mouse, commonly used for genetic, toxicology and pharmacology research. However, training is expected to be successful in other strains, such as the C57/BL6 which have previously been shown to be receptive to habituation protocols (Marcotte et al., 2021; Lindhardt et al., 2022). We suggest that general training and gentling protocols, during acclimatization, would reduce stress during experimental situations and therefore improve animal wellbeing. The gentle handling enables whole experiments to be performed with minimum stress placed upon animals and animal handlers, with unrestrained animals. This refinement is also followed by reduction, as better handling during experimental procedures such as dosing, and blood sampling reduces the risk of mistakes at dosing as well as lost samples. Consequently, the number of animals per group could be reduced to a minimum. Furthermore, lowered stress could potentially lead to reduced sample variability, thus requiring less animals while still maintaining statistical power.

Ear scoring is a sensitive, easily observable, and useful tool for assessing lower levels of distress. This method could therefore be an important tool when assessing improvements of animal welfare in 3R projects and method developments.

## Data availability statement

The raw data supporting the conclusions of this article will be made available by the authors, without undue reservation.

## Ethics statement

The animal study was reviewed and approved by Swedish Research Animal Ethics Committee (Stockholms södra djurförsöksetiska nämnd).

## Author contributions

ET designed and conducted the study as well as collected the data. JS, KW, and ET wrote the initial draft of the manuscript. SB performed the final statistical analyses. CB conducted the study. GN designed the scoring system and performed the initial statistical analysis. All authors contributed to the article and approved the submitted version.

## Footnotes

1
https://www.stats.ox.ac.uk/pub/MASS4/

## Supplementary material

Supplementary Figure 1 Sixteen films were displayed twice, once at the start of the session and a second time at the end to evaluate the scoring stability over the day. Data are represented as the sum of categorical scores: the sum of facial scores from all evaluators (7 evaluators for ear and 6 evaluators for eye) for all mice in each treatment group (*n* = 10) and for all time points in each film (start + challenge + post-challenge). **(A,C)** Total scores for each mouse and day are shown at the start and end of the session. **(B,D)** Violin plots show changes in distribution of scores between the start and end of the scoring session.**p* < 0.05.

Supplementary Table 1 Example of facial scoring sheet used in the scoring session.

Supplementary Table 2 Score per mouse (sum of scores from evaluators) separated by treatment group, day, and time point in the film (start, challenge, and post-challenge). Values are presented as mean ± standard deviation.
