# Supplementary material for: Decreased levels of discomfort in repeatedly handled mice during experimental procedures, assessed by facial expressions
Source: Front Behav Neurosci. 2023 Feb 2;17:1109886. doi: 10.3389/fnbeh.2023.1109886 (PMC9978997; doi:10.3389/fnbeh.2023.1109886)
Supplement: Supplementary file 1 [file Presentation_1.PDF]

Supplementary

Facial Scoring Session

Introduction: Seven evaluators were shown video examples of different ear and eye scores, where they could discuss different aspects of the facial expressions and understand how to grade the facial expressions. After the introduction, during the actual scoring time, the scores were no longer allowed to be discussed.

Schedule:

- 28 videos of animal training sessions
- Break
- 40 videos of mice getting subcutaneous injections
- Lunchbreak
- 40 videos of tail vein blood sampling
- Break
- 28 videos of animal training sessions

Examples of Blood sampling

Supplementary video 7 and 8
