# Supplementary figures and images for: Decreased levels of discomfort in repeatedly handled mice during experimental procedures, assessed by facial expressions
Source: Front Behav Neurosci. 2023 Feb 2;17:1109886. doi: 10.3389/fnbeh.2023.1109886 (PMC9978997; doi:10.3389/fnbeh.2023.1109886)

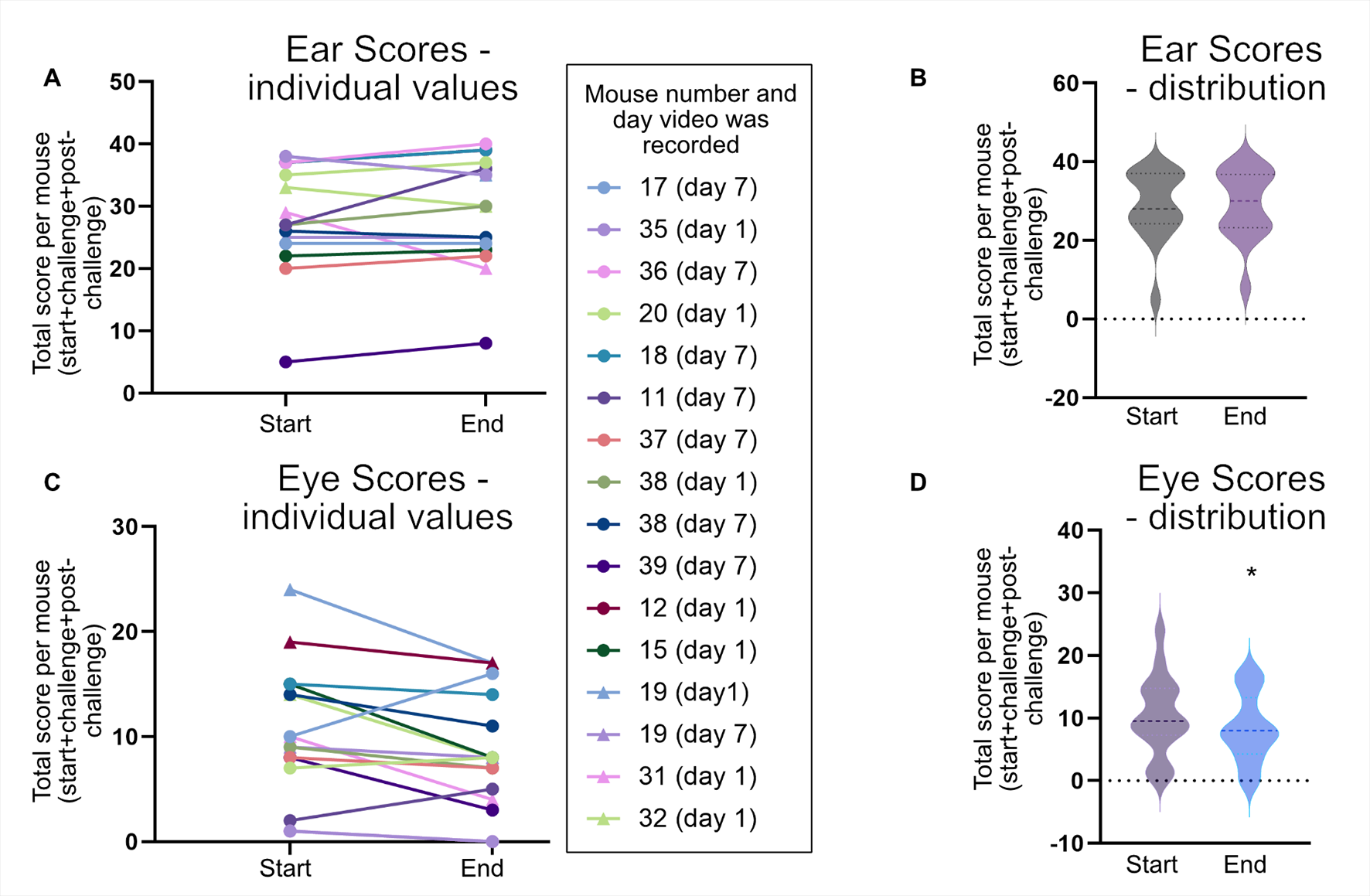

Supplement: Supplementary Figure 1 — Sixteen films were displayed twice, once at the start of the session and a second time at the end to evaluate the scoring stability over the day. Data are represented as the sum of categorical scores: the sum of facial scores from all evaluators (7 evaluators for ear and 6 evaluators for eye) for all mice in each treatment group (n = 10) and for all time points in each film (start + challenge + post-challenge). (A,C) Total scores for each mouse and day are shown at the start and end of the session. (B,D) Violin plots show changes in distribution of scores between the start and end of the scoring session.*p < 0.05. [file Image_1.TIFF]
