# Supplementary material for: Decreased levels of discomfort in repeatedly handled mice during experimental procedures, assessed by facial expressions
Source: Front Behav Neurosci. 2023 Feb 2;17:1109886. doi: 10.3389/fnbeh.2023.1109886 (PMC9978997; doi:10.3389/fnbeh.2023.1109886)
Supplement: Supplementary Table 1 — Example of facial scoring sheet used in the scoring session. [file Table_1.PDF]

**Name:**							
**Video Number**	**Ears**<br>0=Normal ears that stand up<br>1=Ears are slightly towards the head<br>2=ears are right against the head			**Eyes**<br>0=Eyes are completely open<br>1=Eyes are slightly narrowed<br>2=Eyes are very narrowed			**Difficult to score** (x)<br>(Fill in only if it really is difficult to score
	**Start**	**During**	**End**	**Start**	**During**	**End**	
1							
2							
3							
4							
5							
6							
7							
8							
