# Supplementary material for: Decreased levels of discomfort in repeatedly handled mice during experimental procedures, assessed by facial expressions
Source: Front Behav Neurosci. 2023 Feb 2;17:1109886. doi: 10.3389/fnbeh.2023.1109886 (PMC9978997; doi:10.3389/fnbeh.2023.1109886)
Supplement: Supplementary Table 2 — Score per mouse (sum of scores from evaluators) separated by treatment group, day, and time point in the film (start, challenge, and post-challenge). Values are presented as mean ± standard deviation. [file Table_2.PDF]

Measurement day/Procedure	Group	Score - Ear			Score - Eye		
		Start	Challenge	Post-Challenge	Start	Challenge	Post-Challenge
Day 1	Male	9.3 ± 2.5	12.3 ± 2.06	11.3 ± 3.06	3.5 ± 2.76	5.7 ± 2.75	3.3 ± 2.11
	Female	7.4 ± 3.20	11.7 ± 2.41	9.8 ± 3.26	2.9 ± 2.42	5 ± 2.75	2.2 ± 1.48
Day 7	Male Trained	9.4 ± 3.66	10.6 ± 3.27	9.3 ± 4.16	3.2 ± 2.20	3.5 ± 2.32	1 ± 0.82
	Female Trained	4.4 ± 3.78	7.3 ± 4.79	6.9 ± 4.89	1.9 ± 1.60	3.5 ± 2.59	1.5 ± 1.18
Injection	Male control	8 ± 2.49	8.8 ± 2.86	6.7 ± 3.65	0.5 ± 0.71	1.9 ± 1.20	0.5 ± 0.53
	Female control	4.4 ± 3.60	6.5 ± 4.84	4.8 ± 5.22	0.3 ± 0.48	1.9 ± 2.38	0.3 ± 0.67
	Male trained	4.4 ±2.22	6.5 ± 3.14	4.6 ±3.34	0.5 ± 0.71	1.7 ± 1.57	0.7 ± 0.67
	Female trained	2.5 ± 2.01	3.3 ± 2.79	0.6 ± 0.97	0.4 ± 0.52	3.1 ± 3.07	0.6 ± 1.26
Blood Sample	Male control	9.7 ± 3.50	8 ± 4.00	6.1 ± 5.63	0.9 ± 1.60	3.7 ± 2.91	0.7 ± 0.82
	Female control	7.7 ± 5.08	4.7 ± 4.81	2.4 ± 4.6	0.5 ± 0.71	0.5 ± 0.85	0.1 ± 0.32
	Male trained	8.3 ± 3.89	6.3 ± 4.24	3.8 ± 3.88	1.8 ± 1.87	2.2 ± 1.81	0.3 ± 0.48
	Female trained	5.4 ± 3.86	2.9 ± 3.07	0.9 ± 1.60	1.3 ± 1.49	1.5 ± 2.01	0.1 ± 0.32
